# Stomatal decoupling during heatwaves does not alter leaf cooling or recovery in *Quercus ilex* under long-term experimental drought

**DOI:** 10.1093/treephys/tpag108

**Published:** 2026-08-08

**Authors:** Alice Gauthey, Jean-Marc Limousin, Maxwell Bergström, Martin G De Kauwe, Janisse Deluigi, Margaux Didion-Gency, Arianna Milano, Charlotte Grossiord

**Affiliations:** Birmingham Institute of Forest Research (BIFoR), University of Birmingham, B15 2TT, Edgbaston, UK; School of Geography, Earth & Environmental Sciences, University of Birmingham, B15 2TT, Edgbaston, UK; Plant Ecology Research Laboratory (PERL), School of Architecture, Civil and Environmental Engineering (ENAC), EPFL, CH-1015, Lausanne, Switzerland; CEFE, Université de Montpellier, CNRS, EPHE, IRD, 34095, Montpellier, France; Plant Ecology Research Laboratory (PERL), School of Architecture, Civil and Environmental Engineering (ENAC), EPFL, CH-1015, Lausanne, Switzerland; Community Ecology Unit, Swiss Federal Institute for Forest, Snow and Landscape Research (WSL), CH-8903, Birmensdorf, Switzerland; School of Biological Sciences, University of Bristol, Bristol BS8 1TQ, UK; Plant Ecology Research Laboratory (PERL), School of Architecture, Civil and Environmental Engineering (ENAC), EPFL, CH-1015, Lausanne, Switzerland; Community Ecology Unit, Swiss Federal Institute for Forest, Snow and Landscape Research (WSL), CH-8903, Birmensdorf, Switzerland; Plant Ecology Research Laboratory (PERL), School of Architecture, Civil and Environmental Engineering (ENAC), EPFL, CH-1015, Lausanne, Switzerland; Ecological and Forestry Applications Research Center CREAF, 08193 Bellaterra (Cerdanvola del Vallès), Spain; Forest and Soil Ecology Unit, Swiss Federal Institute for Forest, Snow and Landscape Research (WSL), CH-8903 Birmensdorf, Switzerland; Plant Ecology Research Laboratory (PERL), School of Architecture, Civil and Environmental Engineering (ENAC), EPFL, CH-1015, Lausanne, Switzerland; Community Ecology Unit, Swiss Federal Institute for Forest, Snow and Landscape Research (WSL), CH-8903, Birmensdorf, Switzerland; Plant Ecology Research Laboratory (PERL), School of Architecture, Civil and Environmental Engineering (ENAC), EPFL, CH-1015, Lausanne, Switzerland; Community Ecology Unit, Swiss Federal Institute for Forest, Snow and Landscape Research (WSL), CH-8903, Birmensdorf, Switzerland

**Keywords:** acclimation, canopy energy balance, evaporative cooling, leaf thermoregulation, stomatal optimisation modelling

## Abstract

Heatwaves and droughts are intensifying, increasing atmospheric evaporative demand and threatening forest functioning. Rising temperatures drive strong declines in photosynthesis and stomatal conductance, yet some trees maintain transpiration during extreme heat via a phenomenon termed stomatal decoupling. However, its occurrence and significance under long-term drought remain poorly understood. We investigated leaf physiology and canopy temperature in a Mediterranean *Quercus ilex* forest exposed to >20 years of experimental rain exclusion, including during an intense heatwave (>40 °C). Despite similar soil moisture, experimentally droughted trees experienced lower leaf water potential during the summer. Photosynthesis (A_net_) and stomatal conductance (g_s_) were similar across treatments early in the season. During the heatwave, A_net_ declined to zero whilst g_s_ stayed positive, demonstrating strong stomatal decoupling in both drought histories. This decoupling only marginally reduced canopy temperature, which exceeded air temperature by up to 10 °C. Whilst foliar thermal tolerance thresholds (>53 °C) were not surpassed, extensive canopy damage was observed, especially in experimentally droughted trees, and reduced post-heatwave physiological recovery. Overall, *Q. ilex* exhibited stomatal decoupling under extreme heat, but this mechanism provided limited evaporative cooling and did not prevent canopy scorching. Long-term drought mainly influenced recovery capacity rather than the occurrence of decoupling itself.

## Introduction

With climate change, the intensity, frequency and duration of heatwaves are predicted to increase globally (Brown 2020). This projected increase in air temperatures (T_air_) will exacerbate drought impacts by elevating vapour pressure deficit (VPD), thereby intensifying evaporative demand and plant water loss (Grossiord et al. 2020), which contributes to widespread canopy scorching and forest mortality during extreme events (Still et al. 2023). Although high temperatures directly impose non-stomatal limitations on photosynthesis, such as destabilizing key enzymes, disrupting the electron transport chain and promoting the accumulation of reactive oxygen species (Kubien and Sage 2008), the stomatal response to temperature itself remains understudied (Mills et al. 2024). Temperature simultaneously drives two opposing forces on stomatal behaviour: rising VPD, associated with higher T_air_, should trigger stomatal closure (Slot et al. 2024), yet increasing vapour concentration in leaf intracellular spaces with temperature may enhance the evaporative gradient and potentially promote stomatal opening (Mills et al. 2024). Understanding the balance of these processes is essential for predicting leaf water use, photosynthesis and thermal regulation under future climates.

One way to mitigate the negative effects of high T_air_ on photosynthetic capacity is by enhancing latent heat flux via evapotranspiration. Indeed, higher transpiration rates can promote evaporative cooling thereby lowering leaf temperature (T_leaf_). This mechanism can be particularly important during periods of high T_air_, helping to maintain T_leaf_ below critical thermal thresholds (Drake et al. 2018, Blasini et al. 2022, Posch et al. 2024). During intense heat (>35 °C), some tree species can maintain significant transpiration rates despite photosynthetic activity being reduced to near zero (Ameye et al. 2012, Urban et al. 2017, Drake et al. 2018, Aparecido et al. 2020, Wang et al. 2026), a phenomenon often referred to as ‘stomatal decoupling’ (e.g., Marchin et al. 2023). For instance, Urban et al. (2017) showed that under experimental conditions of elevated T_air_ and VPD, net photosynthesis (A_net_) was reduced whilst stomatal conductance (g_s_) increased by ~ 40%, in both *Pinus taeda* and *Populus deltoides* × *nigra* saplings, even in periods of drought stress. Similarly, in saplings of four temperate species exposed to controlled VPD and T_air_ rise, g_s_ increased whilst A_net_ decreased after reaching the optimum temperature for photosynthesis (Diao et al. 2024). These studies hypothesized that, because water viscosity and mesophyll conductance increase with elevated temperatures (Mills et al. 2024), the continued supply of water to evaporative sites enhances stomatal aperture.

Decoupling is often hypothesized to support leaf cooling through sustained evapotranspiration, i.e., latent heat dissipation. Although evaporative cooling typically requires adequate soil water, some species in naturally dry sites appear able to sustain transpiration during extreme heat and soil drought by drawing on deep rooting systems or internal water storage (Salomón et al. 2017, Drake et al. 2018, Aparecido et al. 2020, Bachofen et al. 2025). However, the extent to which decoupling occurs in natural forests, especially under long-term soil drought, remains unclear. Furthermore, ecosystem-scale evidence of stomatal decoupling is mixed (De Kauwe et al. 2019, Krich et al. 2022), with large variability across biomes, species and climatic conditions.

To represent these heat fluxes at large scale, modelling approaches often rely on stomatal optimization models. Yet, a critical challenge is that most stomatal optimization models, particularly those used in land surface models, struggle to represent stomatal behaviour under high temperature. Models based on the Medlyn et al. (2011, 2013) optimization framework assume that stomata maximize carbon gain relative to water loss and predict stomatal conductance (g_s_) from net photosynthesis (A_net_), VPD and atmospheric CO_2_. As a result, these models implicitly assume tight coupling between assimilation and conductance. When A_net_ collapses at high temperature, optimization theory forces g_s_ towards zero, preventing the model from simulating the positive g_s_ characteristic of stomatal decoupling. This leads to a systematic underestimation of transpiration and overestimation of leaf temperature during heatwaves, with important consequences for energy balance modelling. Because canopy temperature predictions in land surface models are directly linked to simulated latent heat flux, any misrepresentation of g_s_ at high temperatures can generate large errors in leaf and canopy temperature, thermal safety margins, sensible heat fluxes and ultimately predictions of heat-related tree mortality. Whilst some work showed that incorporating leaf temperature response could improve model outputs (Chen et al. 2025), model limitations become particularly relevant in regions increasingly exposed to compound hot–drought events, such as Mediterranean forests (Russo et al. 2019, Ruffault et al. 2020).

Drought acclimation may further modify these dynamics. Long-term water limitation is known to alter stomatal density (e.g., Didion-Gency et al. 2024), sensitivity to VPD (e.g., Grossiord et al. 2017, 2018), leaf area (e.g., Limousin et al. 2009, Mas et al. 2024), rooting depth (e.g., Bachofen et al. 2024) and hydraulic safety margins (Garcia et al. 2021, Posch et al. 2024), potentially shifting how trees balance transpiration and carbon uptake during heat. Acclimated trees may maintain residual transpiration (i.e., residual g_s_) under higher hydraulic stress (Limousin et al. 2013, Kruse et al. 2019) or greater sensitivity to VPD (Mediavilla and Escudero 2004), which could either enhance or suppress stomatal decoupling depending on trait adjustments. Yet empirical evidence remains limited, and the extent to which drought history shapes decoupling and its consequences for canopy temperature remains unresolved.

In this study, we aim to understand (i) if stomatal decoupling naturally occurs in *Quercus ilex* under extreme temperatures (>40 °C), (ii) whether chronic drought plays a role in stomatal decoupling and sensitivity to temperature, and (iii) if decoupling was able to induce leaf cooling compared with energy balance expectations using the stomatal optimization framework. The work was conducted in a 20-year rainfall manipulation experiment in a natural *Q. ilex* forest in southern France. We hypothesize that trees growing under ambient control conditions may be exhibiting a more substantial degree of stomatal decoupling during hot periods because of improved water access compared with those exposed to chronic drought. We also predicted that stomatal decoupling could help decrease leaf temperature during hot periods, although this mechanism cannot be captured by current modelling approaches, as they prescribe a coupled relationship between photosynthesis and stomatal conductance.

## Materials and methods

### Experimental site

All measurements were collected at the forest of Puéchabon in Southern France, located 35 km northwest of Montpellier (43°44′29″N; 3°35′46″E, 270 m a.s.l.). The site is dominated by the evergreen Holm oak (*Q. ilex*) with a mean tree height of 5.5 m, a stand density of 4150 stems ha^−1^, a basal area of 27.4 m^2^ ha^−1^, and a leaf area index (LAI) of 2.2 m^2^ m^−2^. The soil is a silty clay loam with a 75–90% rock fraction and a limestone bed. The climate is Mediterranean, characterized by warm and rainy winters and hot and dry summers. The mean annual temperature is 13.5 °C, and the mean annual precipitation is 930 mm (between 1988 and 2017) (Limousin et al. 2009, Ruffault et al. 2023, Heinrich et al. 2025).

In 2003, a partial rainfall exclusion system was set up in the forest using polyvinyl chloride (PVC) gutters situated 1.5–2 m above ground so as to cover 33% of the ground area, and removing ~27% of incoming precipitation after accounting for stemflow. This plot is referred to hereafter as ‘experimental drought’. The ambient plot (i.e., no manipulation) was set up 50 m from the droughted plot in the same forest. This plot is referred to hereafter as ‘control’. Both experimental drought and control plots were equipped with scaffolds, allowing measurements on the top, sun-exposed part of the canopy. More details about the experimental site can be found in Limousin et al. (2009).

Both plots are equipped in their centre with three soil moisture probes (ML2X ThetaProbe; Delta-T Devices Ltd, Burwell, UK) and with six reflectometers (CS616-L Water Content Reflectometers; Campbell Scientific, Logan, UT, USA) installed in the 0–30 cm soil layer. Soil water content was measured over the first 30 cm of soil. The meteorological station is in a forest gap, 100 m away from the experimental drought plot, and recorded air temperature (MP100 sensor; Rotronic, Bassersdorf, Switzerland) and solar radiation (SKS1110; Skye Instruments, Llandrindod Wells, UK) at 2 m above ground (see Figure S1 available as Supplementary Data at *Tree Physiology* Online). Precipitation was measured with a tipping bucket rain gauge (ARG100; Environmental Measurements, Sunderland, UK) situated 1 m aboveground.

### Measurement campaigns

All measurements (detailed below) were carried out over one day for each treatment and repeated three times over the summer 2023 (July, August and September), to encompass a variation of summer temperature and soil water content (see Figure S1 available as Supplementary Data at *Tree Physiology* Online). During the August campaign, the trees experienced the second highest temperatures ever recorded (41.4 °C) at this site (record of 43.4 °C in 2019), leading to a maximum VPD of 6.5 kPa (23 August at 13:30 h). These campaigns are categorized in the text as follow:

- July, ‘cool-dry’ with average daily air temperature (T_air_mean_) of 25.3 °C ± 0.6 and average daily soil water content (SWC_mean_) of 12% ± 0.04;
- August, ‘hot-dry’ with T_air_mean_ = 31.8 °C ± 0.9 and SWC_mean_ =10.1% ± 0.03;
- September, ‘cool-wet’ with T_air_mean_ = 22.3 °C ± 0.9 and SWC_mean_ = 19.1% ± 0.07.

Moreover, August, was characterized as a ‘heatwave’ (defined as including at least one day with one of highest T_air_ ever recorded at the site and four consecutive days where maximum T_air_ > 38 °C), in contrast to the ‘cool’ conditions observed in July and September.

### Leaf water potential

During each campaign, leaf water potential (Ψ_l_) was measured every 2 h from predawn to dusk. For each treatment (i.e., experimental drought and control), eight sun-exposed leaves from different tree individuals (*n* = 8 trees per treatment) were collected at each time and Ψ_l_ was measured using a Scholander-type pressure chamber (Model 1000, PMS Instrument Co., Albany, OR, USA).

### Physiological measurements

Net photosynthesis (A_net_, μmol m^−2^ s^−1^), stomatal conductance (g_s_, mol m^−2^ s^−1^) and transpiration rate (E, mol m^−2^ s^−1^) were measured on the same trees, but a different set of leaves used for Ψ_l_. We measured gas exchange on sun-exposed leaves from the top of the canopy (that leafed out earlier the same year). On the same leaves set, A_net_, g_s_ and E were measured every 2–3 h from sunrise to sunset, using a portable infrared gas analyzer (LI-6800, LI-COR Inc., Lincoln, NE, USA) equipped with a 2 cm^2^ leaf cuvette and after maintaining a steady state rate for at least 2 min. At each measurement, cuvette conditions were set to ambient temperature and humidity (measured separately with a portable RS PRO RS-91 Hygrometer, RS Components Ltd Northants, UK), and ambient light (measured with a quantum sensor attached to the LI-6800 head), and with a reference CO_2_ of 420 p.p.m.

During cool-wet (September) and hot-dry (August) conditions, *A/C_i_* curves (A_net_ vs internal CO_2_ concentrations *C_i_*) were measured using a LI-6800 on sunlit canopy leaves on the same eight individual trees per treatment used for diurnal measurements. Using the *plantecophys* R package, we fitted non-linear curves to estimate the maximum carboxylation rate (V_Cmax_) and the maximum electron transport rate (J_max_) using the same approach as Didion-Gency et al. (2022). As drought and control treatment exhibited similar values, both treatments were pooled for clarity.

Leaf specific conductance (K_L_, mmol m^−2^ s^−1^ MPa^−1^) was calculated as (Duursma et al. 2008):


(1) \begin{equation*} {K}_L=\frac{E}{\varPsi_l} \end{equation*}

with E the transpiration rate (mmol m^−2^ s^−1^) and ⊗Ψ_l_ the difference between predawn and midday leaf water potential (-MPa). To ensure that E is at a steady state with stem sapflow, we only used averaged E values between 11:00 and 14:00 h.

In this study, `stomatal decoupling' was defined as the absence of a strong positive relationship (‘direct coupling’) between A_net_ and g_s_, such that A_net_ was low, zero or negative whilst g_s_ remained positive (Marchin et al. 2023).

### Canopy temperature

Canopy temperature measurements and analysis were carried out following Gauthey et al. (2024*a*, *b*). Briefly, during each campaign, canopy temperature (T_canopy_) was measured on six to eight trees per plot using a drone equipped with an infrared camera. Due to technical issues, T_canopy_ was measured with a different set of instruments in July/August and September. Thermal images were obtained as follows: in July, an uncooled infrared sensor (model DJI Zenmuse XT2) measuring between 7.5 and 13.5 μm with a 640 × 512 pixel resolution was mounted on a DJI M210 RTK V2 drone (DJI Technology Co., Ltd, Shenzhen, China); and in August and September, an uncooled thermal camera (model DJI Zenmuse H20T) measuring between 8 and 14 μm with a 640 × 512 pixel resolution was mounted on a DJI M300 RTK drone (DJI Technology Co., Ltd, Shenzhen, China). Drone flights were executed at regular intervals of time and simultaneously with physiological measurements (i.e., 5–10 flights a day). For each campaign, one full day with clear sky and low wind speed was spent per plot. Thermal images were corrected using a custom-built, black-painted ventilated reference aluminium plate (see Gauthey et al. 2024*a*, *b*). Images were converted to Tagged Image File Format (TIFF) using Forward-Looking Infrared (FLIR) Research Studio V 2.0 software (FLIR Systems Inc., North Billerica, MA, USA), georeferenced, geometrically calibrated and assembled into orthomosaics using Agisoft PhotoScan (Agisoft LLC, St Petersburg, Russia). Assembled single tree canopies were identified using QGIS (QGIS Development Team 2022), and T_canopy_ was measured for each tree canopy as an average pixel value.

The difference in temperature (T_diff_) was calculated as follow:


(2) \begin{equation*} {T}_{\mathrm{diff}}^{i,t}={T}_{\mathrm{canopy}}^{i,t}-{T}_{\mathrm{air}}^{t} \end{equation*}

where T_canopy_ is the canopy temperature (from IR images) for tree *i* and T_air_ is the air temperature (from the meteo station temperature sensor) at time *t*.

### Leaf critical thermal thresholds

At the beginning of our July campaign, we collected one leaf from five different trees from each treatment (i.e., experimental drought and control). Leaves were rinsed with deionized water and rectangular segment (1 cm × 3 cm) were cut out. Using a PlanTherm (PSI, Drásov, Czech Republic), the leaf segment was inserted into a glass test tube and submerged with deionized water. They were then submitted to a gradual increase in temperature (2 °C per minute from 30 °C to 80 °C) and measurements of conductivity (*cond*) and minimum chlorophyll fluorescence (*f0*) were taken at regular time intervals. From these curves (*cond* and *f0* vs temperature), the temperatures at which cell membrane thermostability was exceeded (T_cond_) and the temperature at which the point of maximal acceleration of the chlorophyll fluorescence curve (Ilík et al. 2018) (T_F_) were recorded.

### Statistical analysis

All statistical analysis were done using R (RStudio Team 2015) v. 2025.05.1 + 513.

Diurnal variations of Ψ_l_, A_net_, g_s_ and T_diff_ were analysed for each month separately with a linear mixed effect model using the *nlme* package, with ‘time of day’ included as a random factor to account for repeated measurements. Seasonal differences were analysed for each treatment separately using the same model structure, with month as a fixed effect and repeated measurements on the same leaf included as a random factor. Similarly, we used a linear model followed by an anova to measure difference in T_F_ and T_cond_. *A/C_i_* curves were fitted using the *plantecophys* package and both V_cmax_ and J_max_ were extracted.

### Stomatal optimization and energy balance modelling

To understand whether stomatal decoupling occurred during the measurement period, we analysed the relationship between A_net_ and g_s_ during ‘cool-dry’ (July), ‘cool-wet’ (September) and ‘hot-dry’ (August) conditions. To highlight the effect of temperature/VPD, we merged the cool-dry and cool-wet events (referred to subsequently as ‘cool’ events).

We then used the stomatal optimization model following Medlyn et al. (2011):


(3) \begin{equation*} {g}_s\cong{g}_0+1.6\ast \left(1+\frac{g_1}{\sqrt{VPD_{leaf}}}\right)\ast \frac{A_{net}}{C_a} \end{equation*}

where g_0_ (mol m^−2^ s^−1^) is the residual g_s_ when photosynthesis reaches zero, g_1_ (kPa^0.5^) represents the sensitivity of the conductance to the assimilation rate (‘slope’), VPD_leaf_ the leaf-to-air vapour pressure deficit (kPa), A_net_ (μmol m^−2^ s^−1^) the net photosynthesis and C_a_ (μmol mol^−1^) the atmospheric CO_2_ concentration. g_1_ was fitted with the *plantecophys* package (Duursma 2015). Because we did not have enough measurements at zero photosynthesis to accurately determine g_0_, we fitted g_0_ = 0. We then used the model to predict g_s_ and compared the predictions with measured values from the field separately for the control and the experimental drought treatments. To measure model accuracy, we calculated the root mean squared error (RMSE) of predicted values for both ‘cool’ and ‘hot-dry’ conditions (control and experimental drought treatments). The lower the RMSE, the better the model is predicted to perform.

To understand whether stomatal decoupling impacted leaf temperature (T_leaf_), we modelled T_leaf_ using the *plantecophys* package (Duursma 2015) using the leaf’s energy balance. We modelled T_leaf_ based on both measured g_s_ (T_leaf_measured_gs_) and using modelled g_s_ (T_leaf_modelled_gs_) (see previous paragraph). We hypothesized that if stomatal decoupling contributes to leaf evaporative cooling, then the model, which assumes strong coupling between A_net_ and g_s_, will overestimate T_leaf_ based on the modelled g_s_.

## Results

### Drought treatment

Across the whole growing season, there were no differences in soil moisture between the experimental and control plots (*P*-value = 0.447), suggesting that long-term acclimation allowed chronically drought-exposed trees to mitigate soil moisture reductions (see Figure S1 available as Supplementary Data at *Tree Physiology* Online). This could be explained by various adjustments, including a shift in rooting depth, altered stomatal sensitivity or reduced canopy size (Limousin et al. 2009, 2022, Misson et al. 2010, Martin-StPaul et al. 2013, Moreno et al. 2024). It is important to note that 2023 was a particularly dry year, even in the control treatment, and that the long-term effect of the drought treatment could be observed in the previous years (Limousin et al. 2022).

### Diurnal and seasonal dynamics of carbon and water exchange

**Figure 1:**
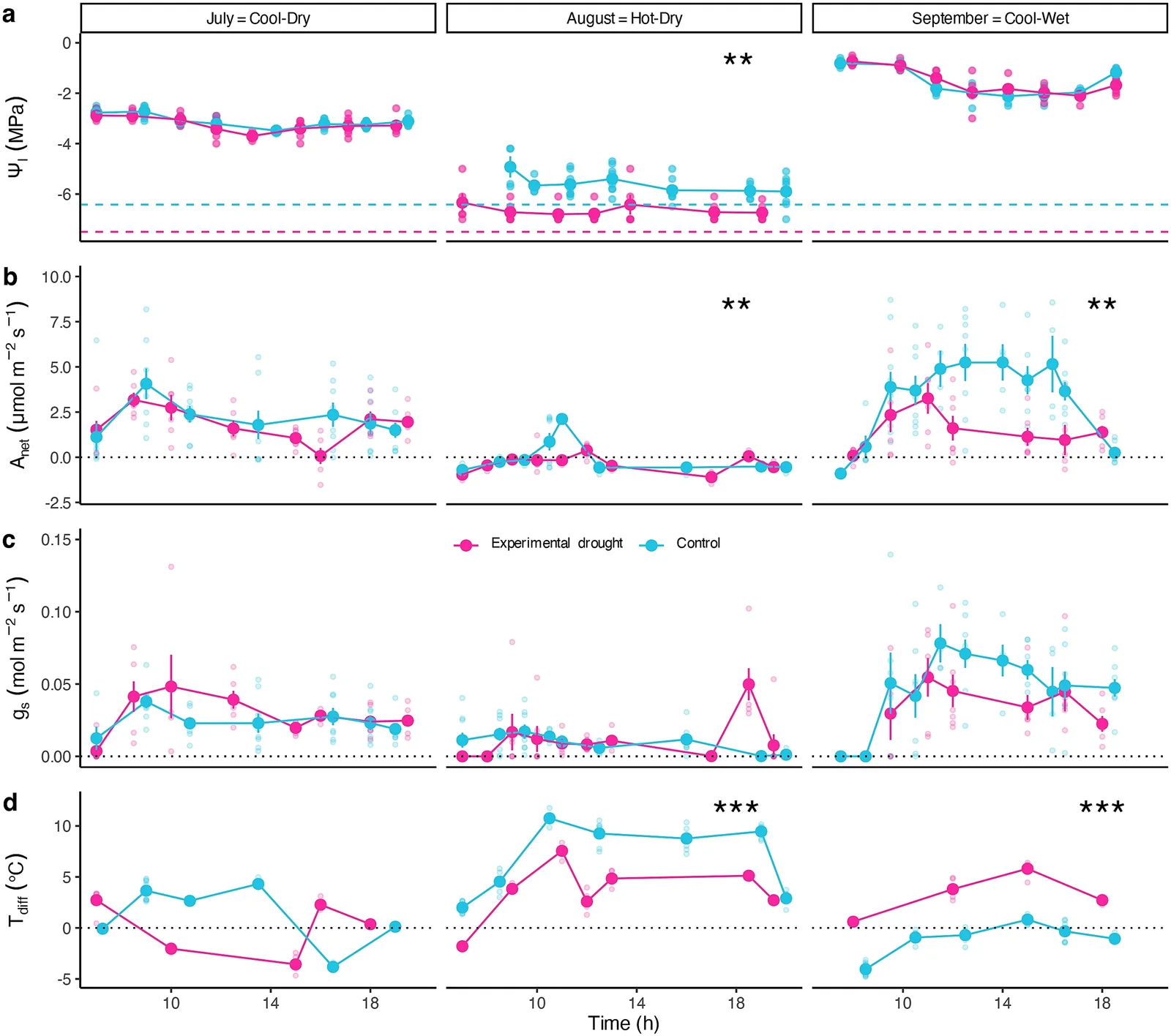
Diurnal dynamics of (a) leaf water potential (Ψ_l_, MPa), (b) net photosynthesis (A_net_, μmol m^−2^ s^−1^), (c) stomatal conductance (g_s_, mol m^−2^ s^−1^) and (d) the difference between canopy temperature and air temperature (T_diff_, °C) in the experimentally droughted (pink) and control (blue) plots during the three measurement campaigns (i.e., July, August and September). Large points represent the average of individual measurements (*n* = 6). The dashed lines in (a) represent the Ψ_50_ (i.e., the stem water potential at which 50% of the hydraulic conductivity is lost) in *Q. ilex* measured at the same site at the start of summer 2019 (i.e., −7.5 MPa and − 6.42 MPa in droughted and control trees, respectively; Moreno et al. 2024); dotted lines in (b), (c) and (d) indicate y = 0. The stars represent the significant differences between treatments for each month (^**^P = 0.01 and ^***^P <0.001).

During cooler conditions (pooled July and September), there was no significant difference in daily average Ψ_l_ between experimentally droughted and control trees (−2.4 and −2.3 MPa, respectively; *P*-value = 0.875). By contrast, during hot-dry conditions in August, daily average Ψ_l_ was significantly lower in experimentally droughted trees (−6.6 MPa) compared with control ones (−5.6 MPa) (*P*-value <0.001) (Figure 1a). After the heatwave, during cool-wet conditions (September), daily average Ψ_l_ in both treatments recovered to high values (−1.6 MPa for both). During the course of the day, Ψ_l_ significantly declined in July and September in both treatments (*P*-value <0.001) whereas this trend was less pronounced in August, with Ψ_l_ exhibiting a relatively flat diurnal pattern.

Daily average A_net_ was significantly lower during hot-dry compared with cool (July and September) conditions (−0.3 μmol m^−2^ s^−1^ vs 2.4 μmol m^−2^ s^−1^, respectively; *P*-value <0.001) (Figure 1b). In September, control trees exhibited a significantly higher A_net_ (2.6 μmol m^−2^ s^−1^) than experimentally droughted ones (1.4 μmol m^−2^ s^−1^; *P*-value <0.05). There was also a significant difference in August (*P*-value <0.05), driven mainly by the higher A_net_ between 10:00 and 12:00 h in control trees (Figure 1b). Similarly, g_s_ was significantly lower during the hot-dry (0.01 mol m^−2^ s^−1^) compared with the cool-wet conditions (0.03 mol m^−2^ s^−1^; *P*-value <0.001) (Figure 1c), but no significant differences were found between treatments.

The difference between canopy and air temperatures (T_diff_) was significantly higher during the hot-dry (+5.3 °C) compared with the cool-wet conditions (+0.6 °C; *P*-value <0.001) (Figure 1d). During the heatwave in August, T_diff_ was significantly higher in control trees, whereas it was significantly lower during cool-wet conditions (i.e., September) compared with experimentally droughted trees.

The maximum rate of carboxylation (V_cmax_) and the maximum rate of electron transport (J_max_) were both significantly lower during the heatwave (V_cmax_ = 1.1 ± 0.2 μmol m^−2^ s^−1^; J_max_ = 6.0 ± 0.2 μmol m^−2^ s^−1^) compared with cooler conditions (V_cmax_ = 13.8 ± 3.1 μmol m^−2^ s^−1^; J_max_ = 27.4 ± 3.1 μmol m^−2^ s^−1^) (*P*-value = 0.03) (see Figure S2a and b available as Supplementary Data at *Tree Physiology* Online). The specific leaf conductance K_L_ was not significantly different between treatments nor months (*P*-value = 0.7695) (see Figure S2c available as Supplementary Data at *Tree Physiology* Online).

### Stomatal decoupling

**Figure 2:**
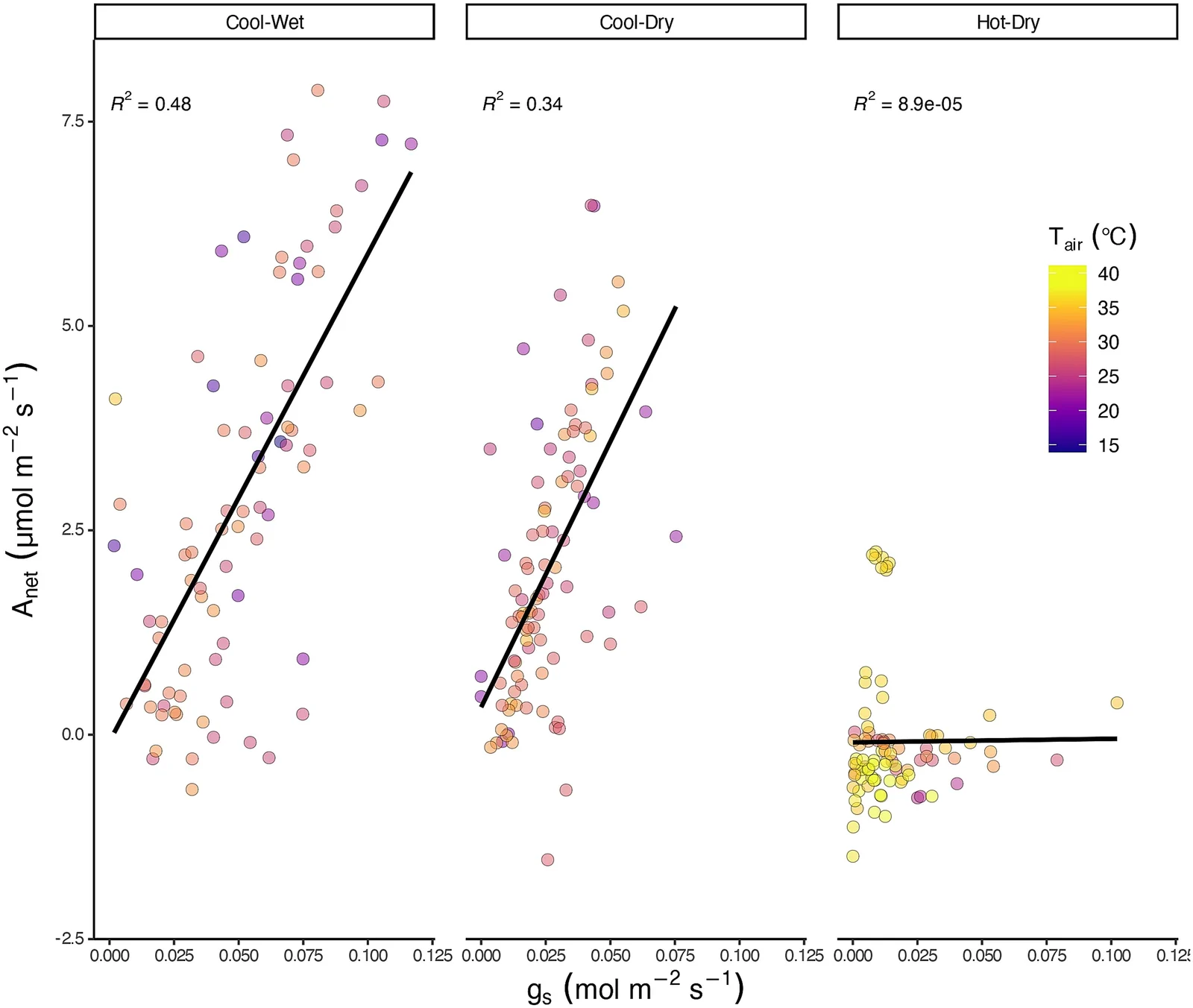
Relationships between net photosynthesis (A_net_) and stomatal conductance (g_s_) for experimentally droughted and control trees pooled together (n = eight trees per treatment) during the three measurement campaigns. Different colours indicate variation in air temperature (T_air_) during the measurements. The *R*^2^ of each linear model is indicated on the top-left side of each panel.

To highlight the effect of the heatwave on the relationship between A_net_ vs g_s_, control and experimentally droughted treatments were pooled for each measurement campaign (see Figure S3 available as Supplementary Data at *Tree Physiology* Online). During ‘cool-wet’ and ‘cool-dry’ conditions, the relationship between A_net_ and g_s_ was significant and positive (*P*-value <0.001), although in cool-dry conditions the slope of the relationship was slightly lower (Figure 2). However, during hotter and drier conditions in August, the relationship between A_net_ and g_s_ was flat and not significantly different from zero (*P*-value = 0.87): A_net_ decreased to values close to or below 0 μmol m^−2^ s^−1^ whilst g_s_ remained positive (up to 0.11 mol m^−2^ s^−1^).

Whilst control and droughted trees exhibited a similar pattern during the heatwave and in ‘cool-wet’ conditions, during ‘cool-dry’ conditions, the relationship between A_net_ vs g_s_ was flatter in experimentally droughted trees (see Figure S3 available as Supplementary Data at *Tree Physiology* Online), indicating that a degree of stomatal decoupling may be triggered by dry conditions.

### Effect of stomatal conductance on canopy temperature and foliar thermal thresholds

**Figure 3:**
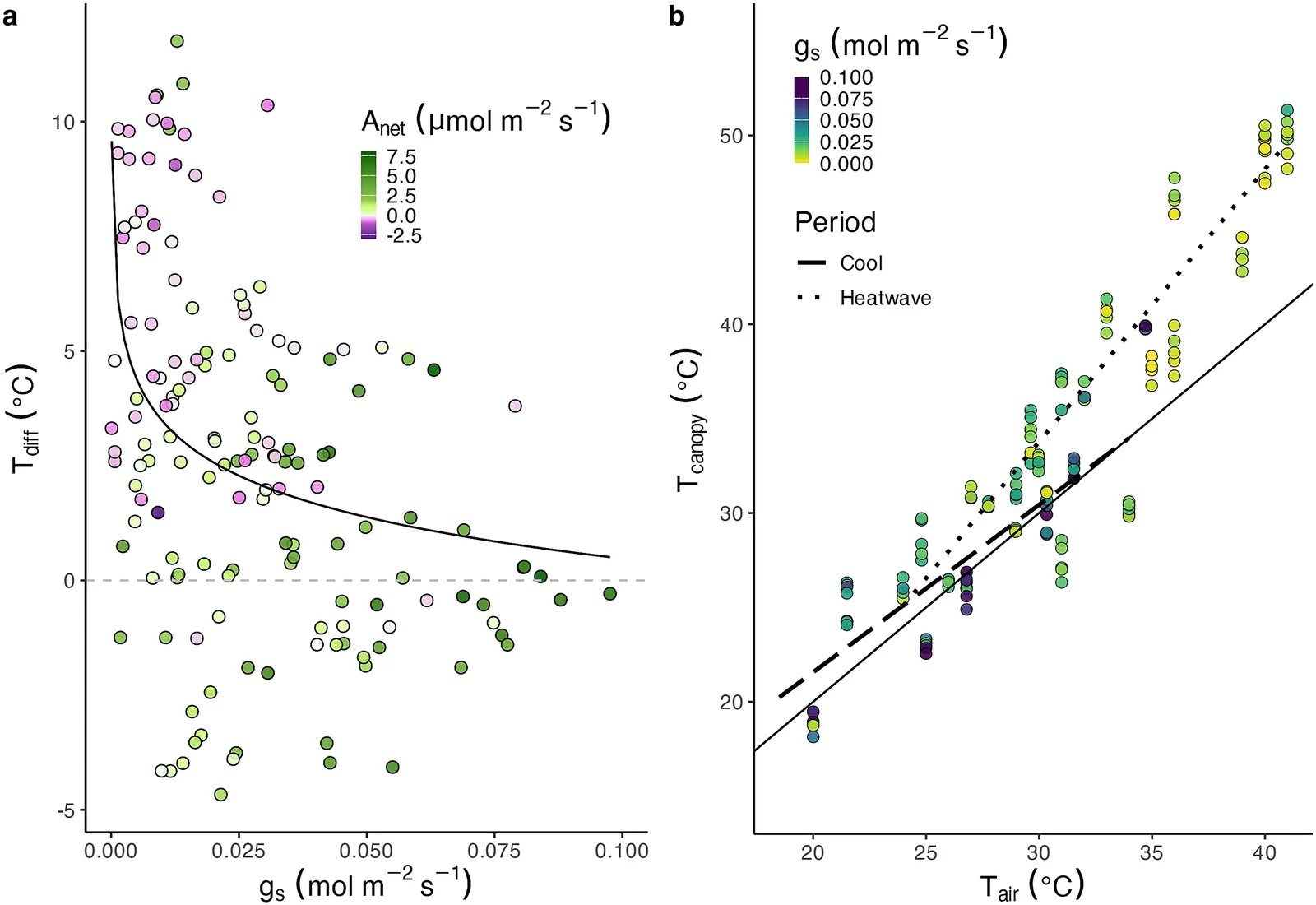
(a) Relationship between the leaf-to-air temperature difference (T_diff_ = T_canopy_ — T_air_) and stomatal conductance (g_s_). Points are individual measurement of T_diff_ (*n* = 8) and are coloured by the values of net photosynthesis (A_net_). The dotted line marks the point where T_diff_ = 0. The full line represents the logarithmic relationship between T_diff_ and g_s_. (b) Relationship between canopy temperature (T_canopy_) and air temperature (T_air_). The solid line represents the 1:1 relationship. The dashed and dotted lines represent the linear relationships between T_canopy_ and T_air_ during cool events (‘cool-wet’ and ‘cool-dry’) and the heatwave, respectively. Points are individual measurements of T_canopy_ (*n* = 8) and are coloured by the values of stomatal conductance (g_s_).

The difference between canopy and air temperature (T_diff_) decreased significantly with increasing g_s_ (*P*-value <0.001), reaching a plateau at higher g_s_ values (Figure 3a). Higher g_s_ was associated with elevated A_net_ and either low (<5 °C) or negative T_diff_. Stomatal decoupling, whereby g_s_ was positive, whilst A_net_ was negative, did not coincide with a decrease in T_canopy_ below T_air_ (Figure 3b). Instead, stomatal decoupling during the heatwave (Figure 2) coincided with T_canopy_ being higher than T_air_ (Figure 3b).

Leaf critical temperature thresholds (T_F_ and T_cond_) were not significantly different between experimentally droughted and control trees (respectively: T_F_ = 54.3 °C and 54.1 °C, *P*-value = 0.8; T_cond_ = 71.6 °C and 70.7 °C, *P*-value = 0.7) (see Figure S4 available as Supplementary Data at *Tree Physiology* Online).

### Modelled vs measured leaf stomatal conductance and temperature

**Figure 4:**
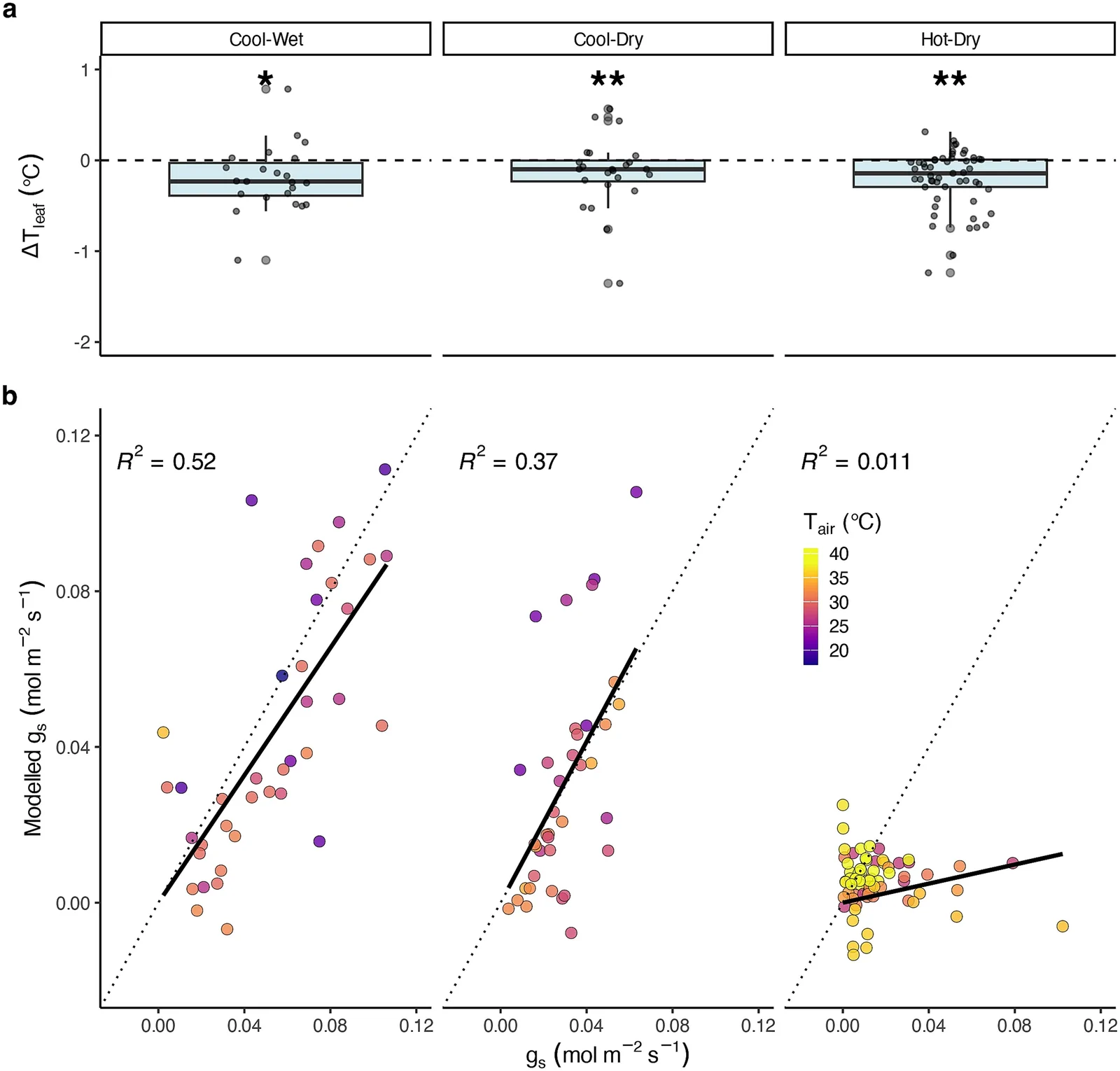
(a) Difference between leaf temperatures obtained with measured g_s_ and modelled g_s_ (⊗_leaf_ = T_leaf_measured_gs_ — T_leaf_modelled_gs_). The stars represent a significant difference to ⊗_leaf_ = 0 for each month (^*^P <0.05 and ^***^  *P* <0.01). (b) Relationship between the modelled g_s_ using the Medlyn stomatal optimization model and measured g_s_ for each period (cool-wet, cool-dry, hot-dry) across a range of air temperature (T_air_, °C). The full line represents the linear regression between the two variables with the *R*^2^ noted for each period. The dotted line represents the 1:1 relationship.

During the two cooler conditions in July and September, the relationship between modelled g_s_ and measured g_s_ was significantly positive with an averaged RMSE of 0.022 mol m^−2^ s^−1^ (*R*^2^ = 0.47) (Figure 4). It was not significantly different from the 1:1 line (*P*-value = 0.06). During the heatwave, the model predicted g_s_ values much lower (<0.004 mol m^−2^ s^−1^) than the measured values (average of 0.01 mol m^−2^ s^−1^) with a RMSE of 0.007 mol m^−2^ s^−1^, and the relationship significantly departed from the 1:1 line (*P*-value <0.001) (Figure 4).

The difference between the T_leaf_ predicted using the measured g_s_ (T_leaf_measured_gs_) and T_leaf_ predicted using the modelled g_s_ (T_leaf_modelled_gs_) (⊗T_leaf_) was not significantly different between cool or heatwave conditions (*P*-value = 0.58) (Figure 4a). Yet, ⊗T_leaf_ values were significantly lower than zero during both cool (−0.21 °C, *P*-value = 0.04) and heatwave conditions (−0.26 °C, *P*-value = 0.01), indicating that T_leaf_modelled_gs_ was significantly higher than T_leaf_measured_gs_. During heatwave conditions, g_s_measured_ was significantly higher than g_s_modelled_ (Figure 4b), likely due to stomatal decoupling (see previous section). Consequently, the negative ⊗T_leaf_ during the heatwave suggests that the higher g_s_measured_ may have contributed to a slight cooling effect on T_leaf_. Although there were no significant differences between g_s_measured_ and g_s_modelled_ during cooler conditions (Figure 4), g_s_measured_ tended to be marginally higher, which may also explain the slightly negative ⊗T_leaf_ observed under cooler conditions. Despite this result, the difference between T_canopy_ and T_leaf_measured_gs_ was lower overall when conditions were cool (July and September), potentially indicating a model overestimation. In comparison, T_leaf_measured_gs_ was closer to measured T_canopy_ than during heatwave conditions (i.e., ⊗ T_canopy_ − T_leaf_measured_gs_ close to 0) (see Figure S5 available as Supplementary Data at *Tree Physiology* Online).

## Discussion

### Stomatal decoupling during hot-dry spells

Our results show that stomatal decoupling was a characteristic response of *Q. ilex* under extreme heat, aligned with reports from other woody species during short periods over 40–41 °C (Urban et al. 2017, Drake et al. 2018, Gauthey et al. 2024*b*). As temperatures exceeded 40 °C, A_net_ collapsed to values near or below 0 μmol m^−2^ s^−1^ whilst g_s_ reached summer maximums of 0.05 and 0.10 mol m^−2^ s^−1^ (in control or experimental droughted trees, respectively) (Figures 1 and 2). In this study, this decoupling occurred despite strong hydraulic stress. Indeed, Ψ_l_ recorded during the heatwave (Figure 1a) dropped below the water potential at turgor loss point (Ψ_TLP_) and close to Ψ_50_ values measured at the same site (i.e., Ψ_TLP_ of −3.7 MPa and − 4.2 MPa, and Ψ_50_ of −6.4 MPa and − 7.5 MPa, for control and droughted trees, respectively; Moreno et al. 2024*b*), which are typically associated with stomatal closure (Brodribb et al. 2003).

Whilst positive stomatal conductance has often been interpreted as a mechanism that enhances evaporative cooling (Urban et al. 2017, Drake et al. 2018, De Kauwe et al. 2019, Muller et al. 2021, Diao et al. 2024, Schuler et al. 2025), our results indicate that the level of g_s_-driven evaporative cooling was insufficient to produce a meaningful reduction in leaf temperature. Despite sustained g_s_, canopy temperatures exceeded air temperature by ~ 10 °C during the heatwave (Figure 3b), and widespread leaf discoloration was observed at the site during the measurements (see Figure S4 available as Supplementary Data at *Tree Physiology* Online). Several scenarios were proposed previously to explain why stomatal behaviour might deviate from optimization (Blonder et al. 2023) and our results bring new evidence to a growing literature showing that, in most forests, sunlit leaf temperatures are rarely downregulated to values lower than air temperatures during the day (Yi et al. 2020, Still et al. 2022, Gauthey et al. 2023, 2024*b*, Manzi et al. 2024) as leaves absorb more solar radiation. Decoupling, therefore, appears insufficient to mitigate thermal stress once VPD, absorbed radiation and tissue temperatures surpass critical thresholds.

Previous research found that when T_air_ exceeded 38 °C, cuticular conductance accounted for almost 100% of total conductance (Slot et al. 2021, Garen and Michaletz 2025), suggesting that stomatal decoupling may not be an active stomatal process. Instead, the positive g_s_ measured may be caused by non-stomatal or residual water loss (Cochard 2021). Indeed, rather than an active cooling strategy, decoupling may represent a physiological ‘last-resort’ state in which minimal transpiration persists through hydraulic or cuticular pathways despite negligible photosynthetic gain, possibly exacerbated by impaired stomatal regulation at high temperatures—for example, due to reduced stomatal control as guard-cell function deteriorates under extreme heat (e.g., membrane instability, e.g., in Scafaro et al. 2021 or loss of turgor responsiveness). In this study, T_air_ was one of the highest ever recorded at this site (42 °C), with VPD reaching over 5.5 kPa between 11:00 and 16:00 h. Stomatal decoupling may slightly delay thermal damage for hours, but sustaining this mechanism could likely accelerate desiccation and compromise post-stress recovery (Cochard 2021). Yet, upon precipitation, Ψ_l_ recovered above pre-heatwave values (Figure 1a), although it did not affect leaf hydraulic conductance as there were no changes in K_L_ across the summer (see Figure S2c available as Supplementary Data at *Tree Physiology* Online). Whilst legacy effects of the heatwave and leaf desiccation could be observed in the canopy damage and the lack of photosynthetic recovery in droughted trees (Figure 1b), we did not measure severe impact on the hydraulic system. Nevertheless, it is important to consider that during the heatwave drought- or VPD-induced embolism (Choat et al. 2012, Schönbeck et al. 2022) may have caused substantial xylem cavitation, thereby reducing K_L_. At the same time, leaf scorching may have reduced the Huber value by September, which would tend to increase K_L_. These opposing effects may have offset one another, resulting in the lack of apparent response of K_L_ (see Figure S2c available as Supplementary Data at *Tree Physiology* Online). Yet, together, these findings may suggest that the photosynthetic limitation under extreme heat and VPD arises primarily from biochemical failure (see Figure S2 available as Supplementary Data at *Tree Physiology* Online), including Rubisco deactivation and declines in electron transport (Scafaro et al. 2023), rather than solely from stomatal closure in *Q. ilex*.

### Mechanisms underpinning stomatal decoupling

The underlying mechanisms of stomatal decoupling remain uncertain. Multiple lines of evidence suggest that stomatal opening is unlikely under the very high VPD (>5.5 kPa) associated with a heatwave (Teskey et al. 2015, Slot et al. 2024). Although high temperatures can induce guard-cell opening via phototropin signalling (Kostaki et al. 2020), this has been observed only under low VPD and white light. The conditions in our study instead favour explanations rooted in (i) biochemical collapse of A_net_, and (ii) temperature-dependent increases in water fluidity enabling continued evaporative flux via stomata or cuticular pathways. Indeed, the fluidity (inverse of viscosity) of water was shown to increase with rising temperatures (Podolsky 1994, Cochard et al. 2000), which could provide a steady water supply to leaf intercellular cavities where water is evaporated at the surface of the leaf as hypothesized by Diao et al. (2024). Additionally, time-lagged responses between biochemical and hydraulic signalling (Joshi et al. 2022) could generate transient g_s_–A_net_ hysteresis, where stomata remain partially open whilst photosynthesis has already ceased. This hysteresis could explain the observed g_s_–A_net_ mismatch and the maintenance of residual transpiration under heat stress. Stored stem or leaf water (Čermák et al. 2007, Scholz et al. 2008, Tian et al. 2019) may further sustain this short-lived transpiration despite declining root water uptake. These mechanisms collectively explain why decoupling persisted across drought treatments and could be observed during cool-dry conditions, and why its magnitude was unrelated to evaporative cooling or canopy temperature.

### Model performance and implications for energy-balance modelling

Similarly to Marchin et al. (2022), the Medlyn stomatal optimization model—as would be the case for any of the standard g_s_ models embedded in land surface models—drastically underestimated g_s_ values during the heatwave (<0.003 mol m^−2^ s^−1^ vs up to 0.10 mol m^−2^ s^−1^ in modelled vs measured values), thus, failing to reproduce decoupling (Figure 4). Our limited understanding of the mechanisms underlying stomatal decoupling constrains the development of more sophisticated modelling approaches. However, our results have provided new insight into the magnitude of this feedback across a *Q. ilex* woodland in Europe. The model’s underestimation of g_s_ had limited implications for canopy cooling. This raises the question of whether stomatal decoupling has a significant impact on the exchange of energy fluxes at the canopy or ecosystem level. Our results suggest it does not: although stomatal decoupling could marginally reduce T_leaf_, its influence on latent heat fluxes was insufficient to significantly reduce canopy temperature (˂0.3 °C; Figure 4a). This limited translation of g_s_-driven cooling at the canopy scale implies that better capturing this process may not be a priority for improving land–atmosphere coupling during heat extremes. It is important to note, however, that the woodland in this study is relatively small in stature (5.5 m tall, LAI ~ 2 m^2^ m^−2^), and that cooling effects due to decoupling may be more visible in denser and higher canopies in other ecosystems (see Posch et al. 2024). Thus, how the magnitude of decoupling feedback varies across different species and along climatic moisture gradients remains to be determined.

### Drought acclimation and stomatal decoupling

The absence of significant differences between treatments during the 2023 growing season was likely influenced by the exceptionally dry conditions this year. Yet, significant differences in leaf water potential observed in most of the preceding years (Limousin et al. 2022) and the significant difference in Ψ_l_ in August 2023 (Figure 1a) indicate that long-term drought treatment remained effective at this site.

Importantly, our results showed that long-term drought acclimation did not alter the occurrence of stomatal decoupling, as both control and experimentally droughted trees exhibited similar g_s_ under extreme heat (Figure 2). This confirms that decoupling in *Q. ilex* is intrinsic and not contingent on high soil moisture, consistent with findings that decoupling may occur even under significant water stress (Marchin et al. 2023). However, drought acclimation strongly modified the consequences of decoupling. During the heatwave, experimentally droughted trees showed higher hydraulic stress (lower Ψ_1_), and displayed lower A_net_ but maintained g_s_ (Figures 1 and 2), possibly reflecting limited stomatal control capacity or altered guard cell sensitivity. Control trees showed greater post-heatwave photosynthetic recovery and reduced canopy damage (personal observation). In contrast, experimentally droughted trees did not recover A_net_ to pre-heatwave values (Figure 1b) despite hydraulics recovery (Figure 1b). Our findings show that despite an array of morphological and physiological adjustments such as lower water potential at turgor loss (Limousin et al. 2022), higher drought resistance (Moreno et al. 2024), reduced A_net_, g_s_ and *J* (Limousin et al. 2010), and decreased LAI (Martin-StPaul et al. 2013), long-term natural adaptation of the control trees to their naturally dry local environment still overrides acclimation mitigation.

Finally, large canopy mortality (i.e., leaf scorching) was reported in the chronically drought-exposed plot (personal observation, see Figure S4 available as Supplementary Data at *Tree Physiology* Online), consistent with lower Ψ_l_ during the heatwave in these trees, suggesting that factors other than SWC, VPD and T_f_ may have played a role in leaf survival (e.g., cuticular wax coverage; Schuster et al. 2016, Bueno et al. 2019). One hypothesis we advance is that the low LAI in the experimental drought plot possibly led to an increase in sensible heat flux (Gauthey et al. 2024*a*), yet exposing lower canopy layers to high VPD and higher light conditions during heat events. By contrast, a higher LAI could result in an increase in whole canopy temperature over extended periods, as trees may impose stricter controls on water-use, heightening the risk of premature leaf discoloration and causing irreversible effects on the photosynthetic machinery (Didion-Gency et al. 2025), impacting photosynthetic recovery. Finally, it is possible that, since thermal thresholds were not exceeded, canopy mortality may be due to drought legacy effects and hydraulic failure instead (Nolan et al. 2021), especially after the already stressful year 2022 in southern France (van der Woude et al. 2023). Overall, we found no evidence that stomatal decoupling during an extreme summer heatwave (i.e., T_air_ > 41 °C) resulted in significant canopy evaporative cooling or that T_leaf_ < T_air_ (Cavaleri 2020, Drake et al. 2020). Although current models substantially underestimate g_s_ during the heatwave, this bias did not lead to a significant change in leaf temperature. Moreover, trees from both drought histories exhibited a similar response to the heatwave, although recovery of gas exchange was more pronounced in control trees.

## Conclusion

Our study demonstrates that stomatal decoupling may be an intrinsic response of *Q. ilex* to extreme heat, occurring irrespective of long-term drought acclimation. Yet, we show that this mechanism provides negligible evaporative cooling and does not enhance post-heatwave recovery. These findings suggest that accurately representing stomatal decoupling is unlikely to substantially improve predictions of canopy energy balance during heat extremes, whilst highlighting the need to better understand the physiological mechanisms underlying this response and its implications for tree resilience under future climate warming.

## Supplementary Material

SI-AG_tpag108

## Data Availability

Data used in this manuscript will be deposited in the Envidat Repository upon acceptance.
