# Supplementary material for: Stomatal decoupling during heatwaves does not alter leaf cooling or recovery in Quercus ilex under long-term experimental drought
Source: Tree Physiol. 2026 Aug 8;46(9):tpag108. doi: 10.1093/treephys/tpag108 (PMC13580088; doi:10.1093/treephys/tpag108)
Supplement: Supplementary file 1 [file si-ag_tpag108.docx]

Article title: **Stomatal decoupling during heatwaves does not alter leaf cooling or recovery in *Quercus ilex* under long-term experimental drought**

Authors: Alice Gauthey, Jean-Marc Limousin, Maxwell Bergström, Martin G. De Kauwe, Janisse Deluigi, Margaux Didion-Gency, Arianna Milano, Charlotte Grossiord

Article acceptance date: Click here to enter a date.

The following Supporting Information is available for this article:

**Fig. S1** Meteorological data

**Fig. S2** Photosynthetic parameters and specific leaf hydraulic conductance

**Fig. S3** Relationship between photosynthesis and stomatal conductance

**Fig. S4** Leaf thermal thresholds

**Fig. S5** Difference between canopy temperature and modelled leaf temperature

**Fig. S1** (a) Daily maximum of vapor pressure deficit (VPD; orange) and temperature (purple) and (b) daily average of soil water content (SWC) in experimental drought (pink) and control (blue) plots. Grey vertical dashed lines represent our three measurement campaigns.

**Fig. S2** (a) Maximum rate of carboxylation (V_cmax_) and (b) maximum rate of electron transport (J_max_) during cool-wet (green, n = 11) and hot-dry (yellow, n = 4) conditions in both treatments combined. (c) Leaf specific conductance during our 3 campaigns et for both treatments (pink= drought and blue=control).

**Fig. S3** Relationships between net photosynthesis (A_net_) and stomatal conductance (g_s_) for experimentally droughted (pink) and control trees (blue)(n = 8 trees per treatment). Different point colours indicate variation in air temperature (T_air_) during the measurements. The *R^2^* of each linear model is indicated.

**Fig. S4** (left) T_F_ and T_cond_ from the Plantherm instrument measured in July for droughted (pink) and control (blue) trees. (right) Pictures of the field site in September 2023, 20 days after the heatwave. The picture depicts a control plot view from the scaffolds.

**Fig. S5** Difference between canopy temperature (T_canopy_) and modelled leaf temperature simulated with measured values of g_s_ (T_leaf_measured_gs_) during ‘cool’ (July and September) and ‘heatwave’ (August) conditions for droughted (pink) and control (blue) trees. Grey dots are default outliers.
